# Bubble entrapment during the recoil of an impacting droplet

**DOI:** 10.1038/s41378-020-0158-y

**Published:** 2020-06-29

**Authors:** Thanh-Vinh Nguyen, Masaaki Ichiki

**Affiliations:** Sensing System Research Center, National Institute of Advanced Industrial Science and Technology (AIST), Japan, 1-2-1 Namiki, Tsukuba-shi, Ibaraki 305-8564 Japan

**Keywords:** Engineering, Physics

## Abstract

When a droplet impacts a (super-)hydrophobic surface, there is a range of Weber numbers within which bubble entrapment will occur during droplet recoil due to closure of the air cavity developed when the droplet spreads out during the impact. In this study, we studied bubble entrapment using a microelectromechanical system (MEMS)-based acoustic sensor fabricated on a substrate. We found that bubble entrapment is followed by an acoustic vibration that can be detected by the sensor. Moreover, the frequency of the vibration is inversely proportional to the radius of the droplet, which indicates that this vibration is the resonant oscillation of the bubble. Therefore, the MEMS-based acoustic sensor can be used not only to detect but also to measure the size of the entrapped bubble. Finally, we demonstrated that it is possible to prevent bubble formation by allowing the air to escape to the underside of the droplet contact area. This can be done by creating through-holes on the substrate or decorating the substrate with sufficiently large textures.

## Microsystems & Nanoengineering Microfluidics: Probing air bubbles trapped in droplets

Researchers in Japan have characterized bubbles of air that form within water droplets when they hit a hydrophobic surface. During impact, an air cavity can form through the center of a droplet, and this can become trapped as bubble when the droplet merges together again. Thanh-Vinh Nguyen and Masaaki Ichiki of the National Institute of Advanced Industrial Science and Technology used a MEMS-based sensor to measure acoustic vibrations during the impact. They found that the frequency of the oscillations is inversely proportional to the size of the entrapped bubble. Furthermore, the team showed that bubble formation can be prevented by changing the topology of the surface to allow the air to escape. These findings will help control the behavior of entrapped bubbles when used for mixing or prevent their formation in applications such as inkjet printing.

## Introduction

**Fig. 1:**
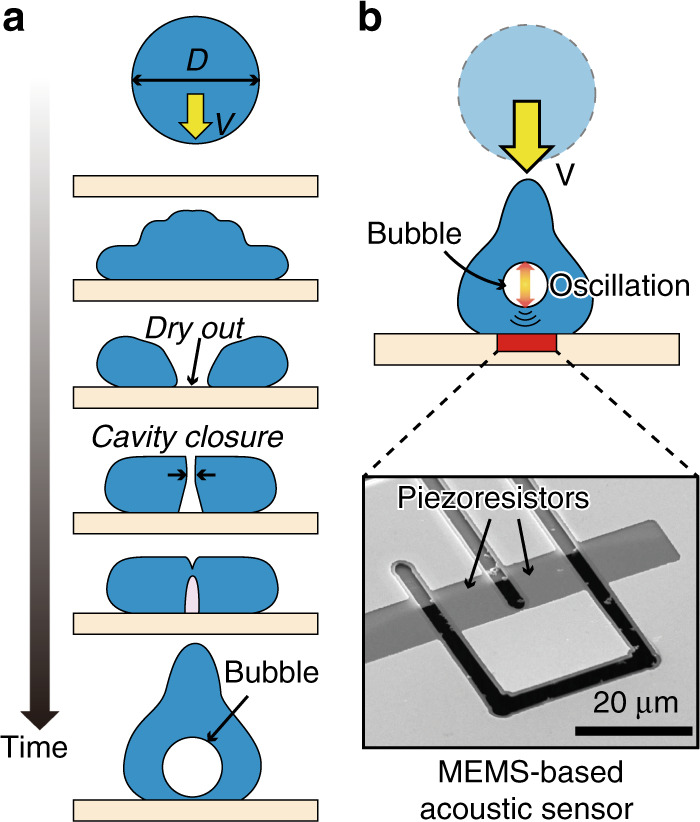
Proposed method using MEMS-based acoustic sensor to study bubble entrapment during the recoil of droplet impact. **a** Mechanism of bubble entrapment during the recoil of droplet impact on a solid substrate. **b** Proposed method to study bubble entrapment

Droplet impact on a rigid substrate has been the topic of various studies because of its fascinating dynamics as well as its important role in a wide range of practical applications^1,2^. When a droplet impacts a solid substrate, bubble entrapment can occur at the beginning of the impact or during the liquid recoil after the spread of the droplet on the substrate. It was shown that prior to the contact of the liquid with the substrate, the droplet slides on a very thin air film sandwiched between the droplet and the substrate^3–9^, and then the liquid/solid contact causes the air film to be trapped, resulting in the formation of a bubble inside the droplet^3–5,10–12^. However, this process is not the only mechanism of impact-induced bubble entrapment. In fact, within a specific range of Weber numbers (defined as We = (*ρDV*^2^)/*σ*, where *ρ*, *σ*, *D*, and *V* are the liquid density, liquid surface tension, droplet diameter, and impact velocity, respectively), the capillary wave occurring on the droplet surface can cause the contact area center to dry out^13^; the recoil liquid then causes the closure of the air cavity, which eventually also leads to bubble entrapment^14–18^, as illustrated in Fig. 1a. This type of bubble entrapment during the recoiling stage of the droplet impact occurs when the contact angle between the liquid and the substrate is high (100 degrees or higher)^15^.

In general, bubble entrapment is undesirable in many applications in which droplet impact is essential, such as in inkjet printing and spray cooling. However, the droplet-entrapped air bubble can also be advantageous in some particular applications; for example, oscillation of the bubble entrapped inside a droplet can be used to enhance droplet mixing^19^. In that sense, droplet impact can be used as an effective method for rapidly creating a bubble inside the droplet. To achieve the best efficiency of bubble oscillation-based droplet mixing, it is necessary to know the size of the bubble to determine the frequency of the external acoustic vibration.

Here, we propose a method using a MEMS-based acoustic sensor for detecting bubble entrapment and simultaneously measuring the bubble size, as shown in Fig. 1b. The bubble entrapment during the recoil phase of the droplet impact is a result of air cavity closure followed by the formation of a singularity jet, which causes the bubble to oscillate at its natural frequency determined by the size of the bubble. Therefore, by directly measuring the bubble oscillation using the MEMS-based acoustic sensor, it is possible to gain quantitative information on the bubble size. On the other hand, because the bubble is formed as the air cavity is sealed, we investigate the possibility of preventing bubble entrapment by letting the air escape, for example, to the underside of the contact area by using substrates that contain through-holes or that are decorated with sufficiently large textures.

## Results and discussion

### Measurement of bubble entrapment

**Fig. 2:**
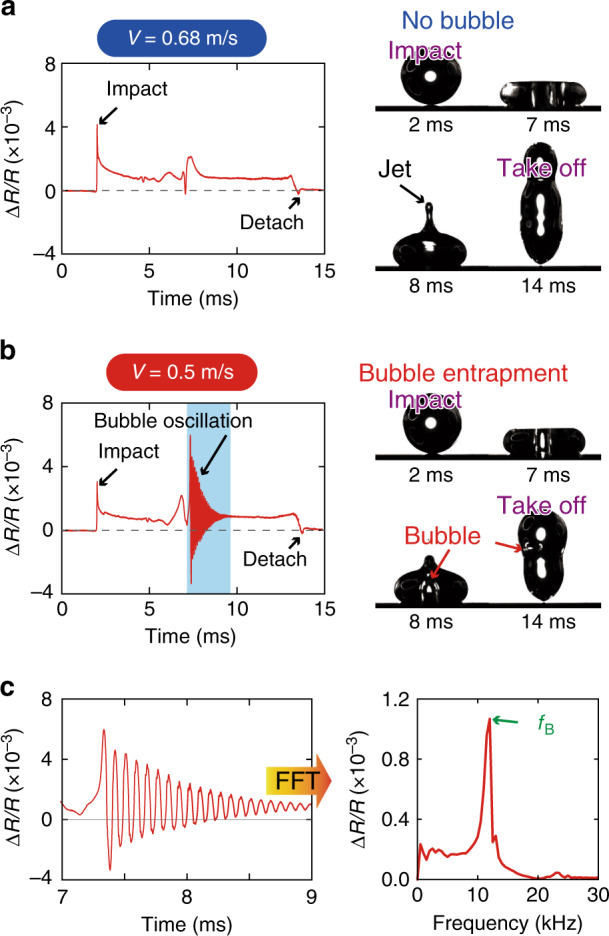
Sensor outputs during the impacts of droplets on the sensor substrate. Responses of the MEMS-based acoustic sensor and images of the high-speed camera (**a**) in the case of no bubble entrapment and (**b**) in the case with bubble entrapment. **c** Acoustic vibration recorded by the sensor signal and its frequency characteristics

Using the fabricated sensor chip, we measured the impacting force and acoustic signal during the impact of water droplets on the sensor chip at different velocities. In each measurement, the location of the droplet was adjusted such that the cantilever was located at the center of the contact area. Figure 2a and (b) shows the responses of the cantilever and snapshots of the high-speed camera in the cases, without bubble entrapment (droplet diameter: 2.2 mm; impact velocity: 0.64 m/s) and with bubble entrapment (droplet diameter: 2.2 mm, impact velocity: 0.50 m/s). The corresponding Supplementary videos S1 and S2 are provided in the Supplementary Information. The occurrence of bubble entrapment can be clearly observed in Supplementary video S2, but not in Supplementary video S1. For both cases, the cantilever’s resistance changed immediately after the droplet hit the sensor chip owing to the pressure caused by the impact. This resistance change was larger for higher impact velocities, as the pressure increases with increasing impact velocity. The most important difference between the signals of the cantilever in the two cases is the vibration presented in the sensor signal when bubble entrapment occurred. A zoomed-in view of the vibration and its frequency characteristics is shown in Fig. 2c (see also Supplementary video S3). From the frequency characteristics, the frequency of the vibration can be obtained as *f*_B_, which was ~12 kHz for the signal shown in Fig. 2c.

### Relationship between the bubble size and the frequency of the acoustic signal detected by the sensor

**Fig. 3:**
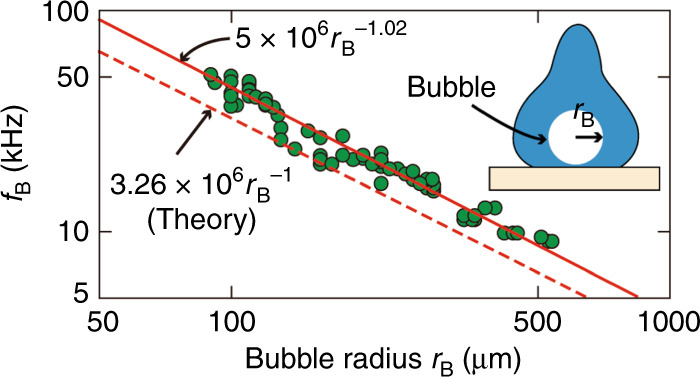
Bubble size vs. frequency of the sensor signal. Relationship between the frequency of the vibration measured by the sensor and the radius of the bubble

The measurement was repeated for different droplet sizes and impact velocities to characterize different sizes of entrapped bubbles. For each entrapped bubble, its radius *R*_B_ was calculated from the images of the high-speed camera. Figure 3 shows the relationship between the radius *R*_B_ of the bubble and the frequency *f*_B_ of the vibration. The solid line in the graph represents the fitting of the data with the function *y* = *ax*^*b*^, where *a* and *b* are the fitting parameters. The result indicates that the frequency of vibration is inversely proportional to the bubble radius, which agrees with the relationship between the natural resonant frequency and the size of the bubble. In fact, neglecting the effect of surface tension and viscosity is possible because the viscosity of water is small and the sizes of the bubbles in our experiment were relatively large (diameter > 100 μm), so that the surface-tension-induced component of the bubble inner pressure becomes sufficiently small in comparison with the ambient pressure. The theoretical relationship between the radius of an ideally spherical bubble and its natural resonant frequency is^20–22^:

1 $$f_{\mathrm{B}} = \frac{1}{{2\pi R_{\mathrm{B}}}}\sqrt {\frac{{3\gamma P}}{\rho }}$$

where *R*_B_, *γ*, *ρ*, and *P* represent the bubble radius, polytropic coefficient, density of the liquid, and the ambient pressure, respectively. Using *γ* = 1.4, *ρ* = 1000 kg/m^3^, and *P* = 10^5^ Pa, we obtain

2 $$f_{\mathrm{B}} =\; \sim \frac{{3.26 \,\times \;10^6}}{{R_{\mathrm{B}}}}$$

where the unit of *R*_B_ is μm. Eq. (2) is represented by the dashed line in the graph of Fig. 3.

The agreement in the scaling law between the measured data and Eq. (2) indicates that the vibration measured by the sensor is indeed the natural frequency of the bubble. The difference in the coefficient parameters of 5 × 10^6^ (measured) and 3.3 × 10^6^ (theoretical) may be due to the error when measuring the radius of the bubbles entrapped inside the droplets from the images; that is, the droplet acts as an optical lens owing to its natural spherical shape, and the bubbles in the captured images appear to be larger than they actually are. Nevertheless, it is important to note that in the bubble application to enhance droplet mixing, the information by the sensor on the natural frequency of the bubble is what is required to determine the frequency of the external acoustic actuation. In other words, the proposed method can be used to rapidly entrap a bubble inside the droplet and then determine the optimized frequency of the acoustic actuation needed to achieve the most efficient mixing.

### Effect of the substrate morphology

**Fig. 4:**
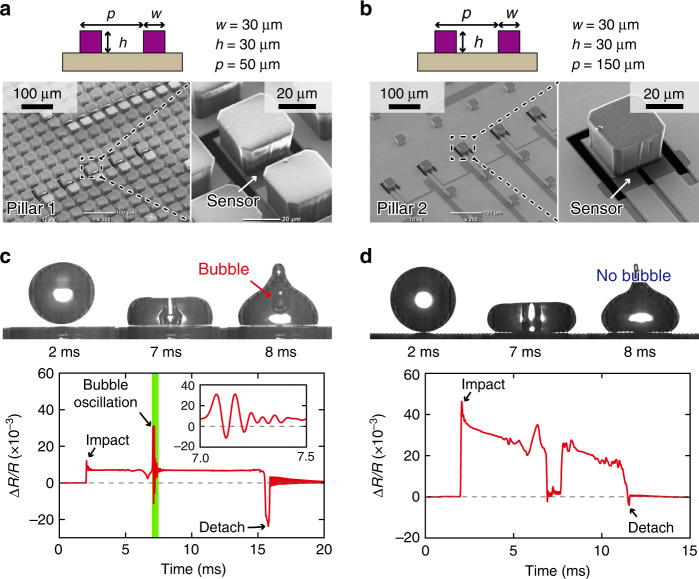
Measurement results for sensors covered with micropillars. **a**, **b** SEM images of fabricated sensor devices with micropillar arrays on their surfaces. **c**, **d** Measurement results for droplets (diameter: 2.2 mm) impacting the sensor devices at a velocity of 0.56 m/s

We investigate the effect of the substrate morphology on the occurrence of bubble entrapment using MEMS sensors whose surfaces are covered with micropillar arrays. SEM images of the fabricated sensor devices (i.e., pillar array 1 and pillar array 2) are depicted in Fig. 4a and b. The dimensions of each micropillar on both sensor devices are 30 μm × 30 μm × 30 μm. Moreover, the pitch *p* of pillar array 1 and pillar array 2 is 50 μm and 150 μm, respectively. For both devices, a MEMS-based sensor was fabricated underneath a micropillar, as shown in the SEM images. The dimensions of the sensor are identical to those of the flat substrate depicted in Fig. 1b.

For the case of droplets with diameters of 2.2 mm and impacting on the sensor devices at a velocity of 0.56 m/s, the impact forces and the images captured using the high-speed camera are presented in Fig. 4c and d for the devices shown in Fig. 4a and b, respectively. The corresponding Supplementary videos S4 and S5 are provided in the Supplementary Information. Based on the images of the high-speed camera, it is evident that despite the identical diameters of the droplets and the identical impact velocities, bubble entrapment occurs in the case of pillar array 1, but not in the case of pillar array 2. It can be observed that over the duration of the impact, the fractional resistance change of the cantilever of pillar array 2 was greater than that of the cantilever of pillar array 1. This difference in the sensor signal amplitudes is caused because pillar array 2 is sparser than pillar array 1; thus, the average force acting on a micropillar of pillar array 2 is stronger than that acting on a micropillar of pillar array 1. In the case of pillar array 1, vibration incorporated with bubble entrapment is observed in the sensor signal, as shown in the inset of the graph depicted in Fig. 4c. In contrast, in the case of pillar array 2, the sensor signal does not exhibit any detectable vibration. Most importantly, the results indicate the possibility of preventing bubble entrapment by alternating the surface morphology of the substrate; this is discussed in detail in the following section.

### Preventing bubble entrapment by surface morphology

**Fig. 5:**
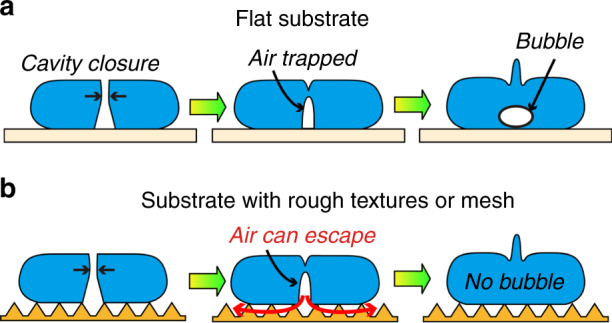
Mechanism for prevention of bubble entrapment by controlling substrate morphology. Conceptual sketches of the air cavity closure and bubble entrapment during the recoil of a droplet impacting (**a**) a flat substrate and (**b**) a substrate with rough textures or through-holes

**Fig. 6:**
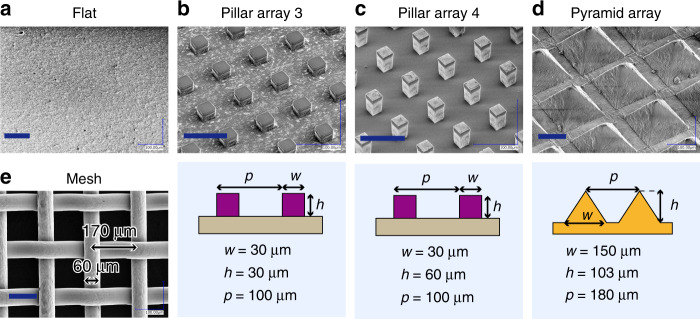
Prepared substrates with different morphologies. SEM images of the substrates prepared for the experiment to investigate the effect of surface morphology on the occurrence of bubble entrapment. The scale bars are 100μm

In this section, we discuss the possibility of using surface textures to control the occurrence of bubble entrapment during the recoil of droplet impact. Because this type of bubble entrapment occurs when the air cavity formed after the spreading droplet is sealed as the liquid recoils to the center, the idea here is to create a pathway for the air inside the cavity to escape so that bubble entrapment can be prevented, as shown in Fig. 5. We tested this idea by investigating the impact of water droplets (diameter *D* = 2.3 mm) on substrates with different surface morphologies: a flat Si wafer, Si wafers featuring micropillar arrays with different pitches and pillar heights, a mesh and an array of pyramid-shaped microstructures. The substrates used in the experiment were pillar array 1 (Fig. 4a), pillar array 2 (Fig. 4b), and the substrates illustrated in Fig. 6. In the experiment, the impact velocity V was systematically varied to change the Weber number within the range of 4–12, which covers the range where bubble entrapment during the recoil phase occurs for a substrate without textures.

**Fig. 7:**
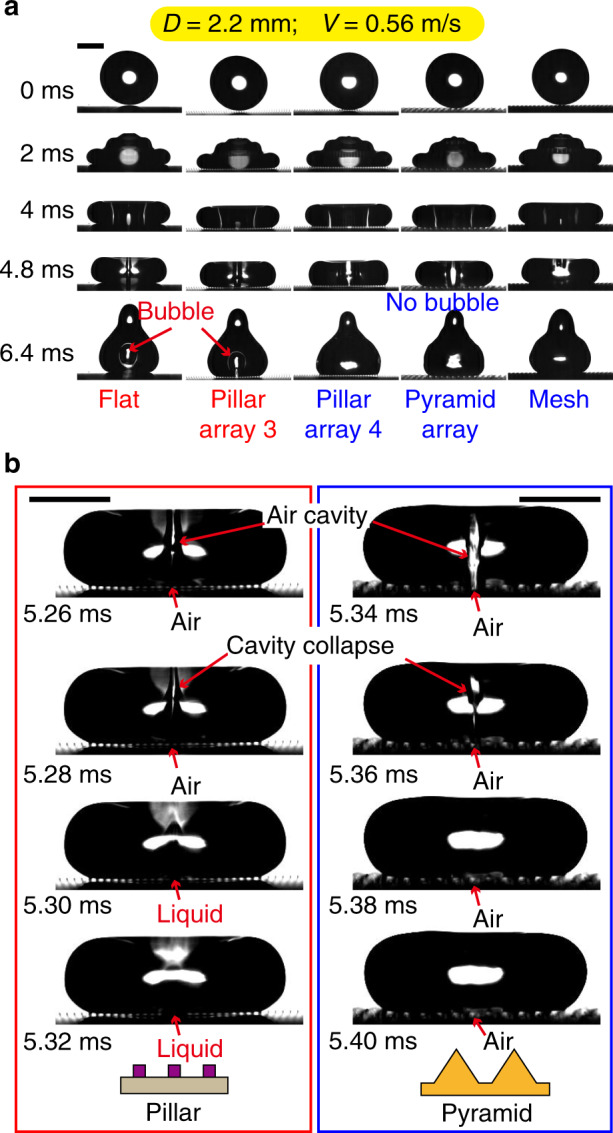
Impacts of droplets on substrates with different morphologies. **a** Impacts of droplets with the same diameters on five substrates with different surface morphologies. The impact velocities were the same: 0.56 m/s. **b** Snapshots showing the cavity closure for the case of the pillar array 3 and a pyramid-shaped microstructure array. Scale bars: 1 mm

The measurement results are shown in Fig. 7. Some snapshots from the high-speed camera showing the behaviors of the droplets impacting the five substrates illustrated in Fig. 6 at the same velocity (0.56 m/s) are shown in Fig. 7a. The corresponding videos (Supplementary videos S6–S10) are provided in the Supplementary Information. The shape evolutions of these droplets exhibit little difference because all the substrates were superhydrophobic and the Weber numbers were the same. By the end of the spreading phase, the air cavities formed and became cylindrical shaped as the liquid recoiled. The difference in the droplet behaviors appeared after the air cavities collapsed: bubble entrapment occurred for the flat substrate (Si wafer) and pillar array 3, but not for pillar array 4, the pyramid-shaped microarray, or the mesh. It is not difficult to understand why bubble entrapment occurred for the Si wafer but not for the mesh; that is, the air inside the cavity could not escape when the Si wafer was used, but it could escape through the through-holes of the mesh. For the micropillar array and the pyramid-shaped microstructure array, the reason for the difference in the impact outcomes is the variation in the texture sizes and density. The results suggest that substrates with sufficiently large textures or large intervals between the textures can prevent bubble entrapment. For instance, as shown in Fig. 4, by increasing the interval of micropillars, it was possible to prevent bubble entrapment. Moreover, increasing the height of the pillar (e.g., pillar array 4 or the pyramid-shaped microstructure array compared with pillar array 3) also enables the prevention of bubble entrapment. The reason for this tendency is that the air inside the collapsing cavity can escape if the textures underneath the droplet are sufficiently large or sparse to maintain the air layer around the textures underneath the air cavity during its closure.

For example, Fig. 7b shows the close-up views of the droplets immediately before and after the moment of cavity closure for pillar array 3 and the pyramid-shaped microstructure array. It can be observed that in the case of pillar array 3, immediately after the closure of the cavity at the top, the area on the contact area below the cavity was filled with liquid (black area in the snapshots), which prohibited the air from escaping. On the other hand, the air gaps surrounding the pyramid-shaped microstructures remained both before and after the closure of the air cavity, thus preventing bubble entrapment.

**Fig. 8:**
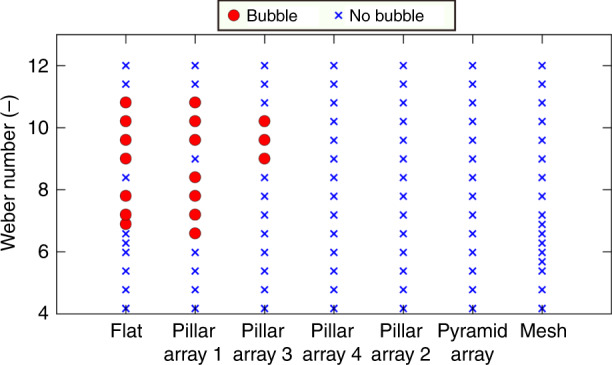
Prevention of bubble entrapment by surface morphology. Effects of the Weber number and surface morphology on the occurrence of bubble entrapment

The effects of the Weber number on the occurrence of bubble entrapment for all substrates are shown in Fig. 8. For a flat Si substrate and pillar array 1 (length: 30 μm, width: 30 μm, height: 30 μm, and pitch: 50 μm), bubble entrapment occurred for Weber numbers in the range of 7–11, whereas pillar array 3 (height: 30 μm and pitch: 100 μm) was capable of preventing bubble entrapment for Weber numbers <8. In contrast, bubble entrapment did not occur when the droplet impacted pillar array 2 (height: 30 μm and pitch: 150 μm), pillar array 4 (height: 60 μm and pitch: 100 μm), the mesh and the pyramid-shaped microstructure array over the entire range of the tested Weber numbers. This result indicates that one can prevent bubble entrapment during droplet recoil after impact using a substrate with sufficiently large or sparse textures or through-holes, which creates pathways for air to escape.

## Conclusion

In this study, we experimentally examined the bubble entrapment induced by air cavity closure during the recoil phase of droplet impact. Using a piezoresistive cantilever fabricated on the substrate, we demonstrated that bubble entrapment is followed by an acoustic signal that can be detected by the cantilever. Therefore, using the cantilever, it is possible to detect bubble entrapment during droplet impact. Moreover, the size of the bubble can also be estimated from the frequency of the acoustic signal measured by the sensor. We also investigated the possibility of preventing bubble entrapment by creating pathways for the air in the closing cavity to escape to the underside of the contact area. The experimental results revealed that bubble entrapment could be prevented by using a substrate with through-holes, such as a mesh, or by decorating the substrate with sufficiently large or sparse textures. These findings not only improve our understanding of the physics of droplet impacts but are also useful in many applications, such as the prevention of bubble entrapment in inkjet printing and the utilization of bubbles in droplet mixing.

## Materials and methods

### MEMS-based acoustic sensor

The sensor used in this study was a MEMS-based piezoresistive cantilever, similar to the sensors that have been developed in our previous studies to measure forces generated by droplets and cells^23–29^. The cantilever was fabricated from a silicon-on-insulator wafer, whose device Si, box, and handle Si layers’ thicknesses were 300 nm, 400 nm, and 300 μm, respectively. The piezoresistive layer was formed by ion implantation, and metals (Cr and Au) were deposited on the device Si layer using vacuum deposition. Next, the cantilever was formed by patterning the metal layers and etching the device Si layer. The piezoresistors were then formed by etching the metal layers at the root of the cantilever. For the fabrication of the sensors with micropillar arrays (Fig. 4a, b), a 30-μm-thick SU-8 nega-photoresist layer was deposited and patterned on the sensor to form the micropillar arrays. Finally, the cantilever was released by etching the handle Si layer and box layer. The size of the cantilever was 35 μm × 30 μm, and the gap surrounding the cantilever was ~1 μm. The size of the sensor chip was 10 mm × 10 mm, and the cantilever was located at the center of the chip. Before the experiment, the sensor chip was coated with hydrophobic nanoparticles to accomplish substrate superhydrophobicity^23,29–31^.

### Substrate preparation

The micropillars, whose sizes were 30 μm × 30 μm × 30 μm, were fabricated by patterning an SU-8 nega-photoresist layer on the Si wafer. The pitch of the micropillar array was 100 μm. The array of pyramid-shaped microstructures was fabricated by casting Polydimethylsiloxane (PDMS) from an anisotropically etched Si wafer. The length of the pyramid’s base, the height of each pyramid, and the interval between two adjacent pyramids were 150, ~103, and 180 μm, respectively. The mesh was purchased from Kyuho Corp. (Osaka, Japan). The diameter of the wire and the pitch of the mesh were 50 μm and 170 μm, respectively. All substrates were coated with hydrophobic nanoparticles through a surface treatment process similar to that used for the sensor chip.

### Measurement setup

The output of the sensor, corresponding to the fractional resistance change of the cantilever, was measured using a Wheatstone bridge circuit connected to an amplifier IC (Texas Instruments Inc., TX, USA, INA 217). The output of the measurement circuit was recorded at a sampling rate of 500,000 samples/s. In the experiment, water droplets were released from the tip of a syringe at different heights to change the impact velocity. The impact of the droplets was captured using a high-speed camera (Photron Ltd., Tokyo, Japan, FASTCAM SA-Z) at frame rates of 100,000 fps for measurements taken using the sensor and 50,000 fps for other measurements.

## Supplementary information

Editorial summary

Video S1

Video S2

Video S3

Video S4

Video S5

Video S6

Video S7

Video S8

Video S9

Video S10
