# Supplementary material for: Bubble entrapment during the recoil of an impacting droplet
Source: Microsyst Nanoeng. 2020 Jun 29;6:36. doi: 10.1038/s41378-020-0158-y (PMC8433192; doi:10.1038/s41378-020-0158-y)
Supplement: Supplementary file 1 — Editorial summary [file 41378_2020_158_MOESM1_ESM.docx]

# *Microsystems & Nanoengineering*

Microfluidics: Probing air bubbles trapped in droplets

Researchers in Japan have characterized bubbles of air that form within water droplets when they hit a hydrophobic surface. During impact, an air cavity can form through the center of a droplet, and this can become trapped as bubble when the droplet merges together again. Thanh-Vinh Nguyen and Masaaki Ichiki of the National Institute of Advanced Industrial Science and Technology used a MEMS-based sensor to measure acoustic vibrations during the impact. They found that the frequency of the oscillations is inversely proportional to the size of the entrapped bubble. Furthermore, the team showed that bubble formation can be prevented by changing the topology of the surface to allow the air to escape. These findings will help control the behavior of entrapped bubbles when used for mixing or prevent their formation in applications such as inkjet printing.

Related article manuscript number: MICRONANO-01115R

Article title: Bubble entrapment during the recoil of an impacting droplet

Corresponding author and affiliation/s: Thanh-Vinh Nguyen, National Institute of Advanced Industrial Science and Technology (NAIST), Japan

**About your Editorial Summary — please read**

**Before approving this Editorial Summary, please carefully check that (1) the summary text lists the correct author(s) and (2) the spelling and order of all author names and affiliations are correct.**

This **Editorial Summary** is based on your manuscript that was recently accepted for publication in *Microsystems & Nanoengineering*. It provides a non-specialist audience with a synopsis of your key research outcomes and conclusions. This value-added service provided by Springer Nature is designed to raise interest in your research across the broader community.

Springer Nature will publish the summary on the journal’s website, and it will be freely available under a under the CC BY licence (Creative Commons Attribution v4.0 International Licence) (see the journal website for details). We encourage you to re-use the summary to bring attention to your research; for example, you can host it on your own website and share it via social-networking platforms. Please attribute the summary to *Microsystems & Nanoengineering* and your article (e.g. by providing a link to your article) and do not make derivatives.

Please note that to maximise the usefulness of these summaries they must follow several stringent guidelines:
-- Spelling, punctuation and style are set according to *Nature* editorial guidelines. As this summary is aimed at non-expert readers, some concepts and technical terms will be simplified.
-- Total length must be no more than 135 words. It is likely that not all points in the paper will be covered.
-- The first sentence must be no more than 280 characters, including spaces, to allow use on microblogging sites.
-- The headline must consist of a brief generic subject identifier followed by a short description. No more than 10 words in total.

Please contact the editorial office ([mems_nano@mail.ie.ac.cn](mailto:mems_nano@mail.ie.ac.cn)) immediately with corrections should you find any factual errors in this Editorial Summary.
